# The Potential Link between Gut Microbiota and IgE-Mediated Food Allergy in Early Life

**DOI:** 10.3390/ijerph10127235

**Published:** 2013-12-16

**Authors:** John Molloy, Katrina Allen, Fiona Collier, Mimi L. K. Tang, Alister C. Ward, Peter Vuillermin

**Affiliations:** 1 Child Health Research Unit, Barwon Health, Geelong, Victoria 3220, Australia; E-Mails: John.molloy@mcri.edu.au (J.M.); FionaC@Barwonhealth.org.au (F.C.); 2 School of Medicine, Deakin University, Waurn Ponds, Victoria 3216, Australia; E-Mail: Alister.ward@deakin.edu.au; 3 Murdoch Children’s Research Institute, Parkville, Victoria 3052, Australia; E-Mails: Katrina.Allen@rch.org.au (K.A.); Mimi.tang@rch.org.au (M.L.K.T.); 4 Department of Allergy and Immunology, Royal Children’s Hospital, Parkville, Victoria 3052, Australia; 5 Department of Paediatrics, University of Melbourne, Parkville, Victoria 3052, Australia

**Keywords:** gut microbiota, immune development, food allergy

## Abstract

There has been a dramatic rise in the prevalence of IgE-mediated food allergy over recent decades, particularly among infants and young children. The cause of this increase is unknown but one putative factor is a change in the composition, richness and balance of the microbiota that colonize the human gut during early infancy. The coevolution of the human gastrointestinal tract and commensal microbiota has resulted in a symbiotic relationship in which gut microbiota play a vital role in early life immune development and function, as well as maintenance of gut wall epithelial integrity. Since IgE mediated food allergy is associated with immune dysregulation and impaired gut epithelial integrity there is substantial interest in the potential link between gut microbiota and food allergy. Although the exact link between gut microbiota and food allergy is yet to be established in humans, recent experimental evidence suggests that specific patterns of gut microbiota colonization may influence the risk and manifestations of food allergy. An understanding of the relationship between gut microbiota and food allergy has the potential to inform both the prevention and treatment of food allergy. In this paper we review the theory and evidence linking gut microbiota and IgE-mediated food allergy in early life. We then consider the implications and challenges for future research, including the techniques of measuring and analyzing gut microbiota, and the types of studies required to advance knowledge in the field.

## 1. Introduction

Food allergy is one of the epidemics of the modern world. Over the last twenty years rates of potentially life threatening reactions to food (anaphylaxis) have steadily risen in the developed world [1]. Such increases have been documented in the United Kingdom, the United States of America (USA) and developed areas of Asia [2,3,4]. Concordantly, in Australia during the 1990’s and 2000’s admission rates for food induced anaphylaxis through the Emergency Departments increased threefold primarily in children less than five years old [5]. Indeed it has been demonstrated in recent years that as many as 8%–10% of one year old infants living in Melbourne Australia have challenge proven IgE-mediated food allergy [6]. In children the commonest reason for anaphylaxis admission is IgE-mediated food allergy [5] and the cause of this epidemic remains elusive. 

The original hygiene hypothesis by Strachan proposed that exposure to infection in early life through larger family sizes was protective against allergic disease [7]. Subsequently the hypothesis has been “revisited” through work on the potential association between nonpathogenic microbial experience, immune development and allergic disease [8,9]. Allergic disease is more prevalent in modern urbanized communities [1] suggesting factors in the modern lifestyle and environment have driven the increase in food allergy. An example is the strong association of early life farm exposure and lower levels of atopic sensitization. This association has been shown in several studies including the GABRIELA and PARSIFAL cohorts [10,11,12]. It appears this early life farm exposure effect is mediated predominantly by microbial experience as children on farms are exposed to diverse microbial environments. It is plausible that such early life exposure modifies the composition of the human microbiome which may explain the association between non-pathogenic microbial experience, immune development and allergic disease. 

Recent advances in the measurement and analysis of the human microbiome have led to a dramatically more detailed understanding of the scale and variation of the gut microbiome [13] and a growing appreciation of the potential importance of the human microbiome in health and disease [14]. Of specific relevance, it has been shown that gut microbiota have a profound effect on gut immune development, barrier function and response to food antigens [15,16]. The human microbiome project has provided vast information on the determinants of the gut microbiota including the opportunity to examine its influence on non-communicable disease processes. There are a range of factors in the modern environment that may be associated with changes to both the microbiome and risk of food allergy, such as mode of delivery, antibiotic exposure, infant feeding practices, farming environment and country of origin.

In this review, we explore the theoretical basis for a relationship between gut microbiota and IgE-mediated food allergy. We then assess the current evidence for the association between specific factors in the modern environment and both: (a) the composition of the gut microbiota and (b) IgE-mediated food allergy in children. Finally, we consider the opportunities and challenges for future research in this area, including a discussion of the various techniques available for measurement and analysis of gut microbiota, as well as the study designs that are required.

## 2. The Theoretical Basis for a Relationship between Gut Microbiota Development and IgE-Mediated Food Allergy

There is a strong theoretical basis for the hypothesis that the composition of the gut microbiome during early life may influence the risk of food allergy. IgE-mediated food allergy is characterized by allergic sensitization and subsequent clinical allergic reaction following antigen re-exposure through the gut. Sensitization is the process where allergen specific T and B cells recognize and respond to an allergen leading to production of allergen specific IgE antibodies. The state of sensitization may or may not be associated with clinical allergic reaction upon re-exposure. The factors that lead to sensitization are poorly understood. One possible mechanism is resistance to or escape from gastric acid or enzymatic digestion. Ingested dietary proteins are generally not presented to the Gut Associated Lymphoid Tissue (GALT) as a result of gastric acid or enzymatic digestion and an intact gut epithelial barrier. Previous experiments on mice have shown that antigens that have bypassed gastric acid digestion through intra-ileal delivery are immunogenic once absorbed whereas antigens that have undergone gastric acid digestion and subsequently are absorbed induce tolerance [17]. Another experiment showed that oral tolerance could be disrupted by feeding mice encapsulated antigens that avoided gastric acid and enzyme digestion [18]. Concordantly, many food allergens, such as egg and peanut, have been shown to be resistant to breakdown by gastric acid [19]. It has also been shown that defects in gut barrier function are associated with an increased risk of allergic sensitization [20]. The presence of sensitization does not always result in clinical allergy. Only 30%–55% of people who are sensitized to food allergens such as egg, milk or peanut are also allergic and will have an allergic reaction upon re-exposure [6]. The factors that determine whether or not an individual who is sensitized also manifests clinical allergy remain unknown. In allergic subjects, allergen-specific IgE is bound to the surface of mast cells via high affinity FcE receptors. On re-exposure, allergen binding to allergen-specific IgE results in crosslinking of surface bound IgE and this in turn stimulates mast cells to release preformed mediators and produce additional pro inflammatory mediators, which together lead to an allergic response. Disruption in early gut microbiota development may alter gut epithelial integrity, affect immune development and potentiate the allergic response. The theoretical basis for this process is discussed below.

### 2.1. Development and Modification of the Gut Microbiome during Early Life

The term gut microbiota describes the total population of bacteria in the gut; there are an estimated 10^13^ to 10^14^ organisms present comprising at least 400 species of bacteria [21,22]. The gut microbiome contains more than 100 times the number of genes in the human genome. Phase 1 of the Human Microbiome Project has demonstrated the dramatic variation in the composition of gut microbiota, both between individuals, and within individuals over time [13] and the implications of such variation are the subject of intense research activity.

The co-evolution of humans and their commensal gut organisms has resulted in an elegant symbiosis in which gut microbiota play a crucial role in the development and maintenance of gut immune and barrier function [15]. If this relationship becomes unbalanced, pathological processes such as immune mediated bowel inflammation, or conversely, sepsis can occur where invasion by opportunistic bacteria may prove fatal to the host. Thus, in order to maintain an effective balance, the immune system has evolved a variety of mechanisms to provide benefits for both host and commensal organisms. For example, tolerance is the ability of the immune system to recognize a harmless antigen and actively suppress an inflammatory response. Given the immense antigenic load presented by gut microbiota, the maintenance of tolerance is crucial to avoid excessive inflammation. The process of immune tolerance is assisted by anatomical isolation of microbiota within the gut lumen [23]. Goblet cells in the epithelial wall produce a mucus barrier that impedes the microbiota from penetrating the inner epithelium [24]. In addition, epithelial cells also produce a range of antimicrobial proteins, belonging to the same family of proteins as defensins that have the capacity to kill bacteria by compromising bacterial cell wall integrity [25]. Furthermore, secretory IgA is produced by B cells induced in the GALT and secreted across the epithelial wall. Gut secretory IgA is thought to be involved in trapping bacteria in the mucus layer preventing pathological invasion [26], thereby maintaining the normal host-microbiota balance [27]. Secretory IgA may also play a role in oral tolerance through the binding of antigens in the gut and preventing systemic uptake of the antigen [28]. In this context it has been shown that in mice sensitized to *β*-lactoglobulin (a protein in milk) intestinal IgA levels are depressed compared to those in mice tolerant to *β*-lactoglobulin [29]; and further, that food allergy rates are higher in children with IgA deficiency [30].

In concert with host factors described above and barrier function, the commensal bacteria occupying the gut are genetically programmed not to express factors that enable cell invasion. Consequently if commensal bacteria happen to invade the epithelium they are quickly phagocytosed by the host [31]. Pathogenic organisms that manage to evade these host barriers to invasion can cause infection. For example, *Campylobacter jejuni* and *Salmonella*, which may result in life threatening illnesses, have flagella that aid invasion and appear resistant to antimicrobial proteins, while other pathogenic organisms are able to downregulate factors that are microbicidal [32,33].

### 2.2. Host Influences on Microbiome Development

The early life colonization of the gut by bacteria essential for normal immune development is affected by factors including mode of delivery, antibiotic exposure, dietary intake and developing *vs*. developed world lifestyle. In addition to environmental factors there is some evidence that host genotype may influence the pattern of gut colonization during early life, which in turn, may predispose to specific gene-environment interactions [34]. For example, in experimental models, inbred mice have more comparable gut microbiota profiles than outbred mice [35]. Concordantly, in a twin study conducted among children aged up to ten years, the fecal microbiota of identical twins, fraternal twins and unrelated individuals were compared [36]. The similarity of gut microbiota patterns was greatest among identical twins. However, a subsequent study among adult twins found no evidence of an association between host genotype and the composition of the gut microbiome [37]. This may reflect difference in the relationship between genotype and the gut microbiome in children as compared to adults, but it may also reflect study differences. In particular, the study among children used temporal temperature gradient gel electrophoresis (TTGE) to characterize the gut microbiota, whereas the study among adults used pyrosequencing, which provides a far more detailed picture of the microbiome.

### 2.3. The Impact of the Gut Microbiome on the Developing Immune System

The role the gut microbiota plays in immune development and acquisition of tolerance has been explored in various immune studies on germ free mice. The mouse is a useful model as ninety nine percent of mouse genes are shared with humans and their gut microbiota is quite similar to the human gut microbiome [38]. Early experiments demonstrated that gut associated lymphoid tissue, comprising organized structures of lymphoid tissue such as Peyer’s patches are poorly developed or absent in germ free mice [39,40]. Subsequent work has shown that germ free mice have reduced numbers and function of (CD4^+^CD25^+^Foxp3^+^) Tregulatory (Treg) cells within the mesenteric lymph nodes and Peyer’s patches, likely representing induced Treg’s (iTreg), which play a key role in the induction of tolerance, providing a putative mechanism for failure of oral tolerance in such mice [41,42,43]. It has been demonstrated that introduction of *Bacteroides fragilis* into the lower gut of germ free mice can redevelop the GALT and induce tolerance but only if done in the neonatal period. The introduction of *Bacteroides fragilis* is associated with induction of Treg’s cells via IL-10 dependent mechanisms which may explain its role in tolerance induction [44].

Allergic disease is, at least in part, related to enhanced responses from Th2 cells [45]. During pregnancy the Th1 immune response of the fetus is suppressed to prevent excessive response to maternal antigens, and as a result, at birth the infant is skewed toward a Th2 response to novel antigens [46]. Th2 cells produce IL-4, IL-5 and IL-13 which aid the development of allergic inflammation. Exposure to gut microbiota shifts this response to development of Th1 cells, which promotes immune tolerance and maintains a Th1/Th2 balance [44,47]. The gut microbiota is one of the environmental signals that promote T-cell maturation [48]. A recent study highlighted that a lack of *Bacteroides* colonization may be associated with a poor Th1 response [49]. The Th1/Th2 balance is not the sole mechanism to maintain immune tolerance, indeed increased numbers of Th1 cells may be associated with established allergic disease [50]. Germ free mice demonstrate a persistent Th2 skew as a result of absent intestinal microbiota.

### 2.4. Experimental Evidence Specifically Linking the Gut Microbiota and Development of Food Allergy

Further experimental work suggests the relationship between gut microbiota and the immune system is interdependent in developing and maintaining tolerance, thus avoiding development of food allergy. IL4ra gain of function mutant mice are particularly susceptible to allergic sensitization. In a recent study by Noval Rivas *et al.* IL4ra mice had altered gut microbiota prior to sensitization compared to allergy resistant mice [16]. The establishment of sensitization in the IL4ra mice was associated with an altered microbial signature suggesting immune influence on the gut microbiota. When gut microbiota from allergic mice was transferred to allergy resistant mice, food allergy could be induced in the previously resistant mice suggesting the microbiota could modify the immune response. Finally when they transferred allergen specific Treg cells into the IL4ra mice the process induced tolerance and suppressed further sensitization attempts. This is consistent with work by Yamashita *et al*. that also showed that transfer of Treg cells from allergen tolerant mice into naïve mice suppressed subsequent attempts at food induced anaphylaxis [51]. 

### 2.5. Direct Evidence that the Composition of the Gut Microbiome Influences the Risk of Allergic Disease in Humans

Compared to the few studies on gut microbiota and food allergy, the relationship between gut microbiota and eczema has been extensively studied, and eczema and food allergy are strongly associated entities. Wang *et al.* demonstrated reduced gut microbial diversity at one week of age in infants who subsequently developed eczema by eighteen months of age [52]. This finding was reproduced in a prospective study of infants determined to be at high risk of allergic disease that showed gut microbial diversity was reduced in the first week of life in children who developed atopic eczema by one year of age [53]. The lack of diversity in both studies was determined using the molecular technique T-RFLP. Abramhasson *et al.* showed using 16S rRNA sequencing that gut microbial diversity was reduced at one month of age in infants who subsequently developed IgE-related eczema with *Bacteroides* species lower in infants with eczema [54]. In each of the three studies discussed above gut microbiota was analyzed at the age of one week or one month each demonstrating reduced microbial diversity prior to the onset of atopic disease, a recognised strength of the studies. In contrast a more recent study by Nylund *et al.*, using DNA microarray as a method of analyzing the microbiota, found greater microbial diversity in infants with atopic eczema at 6 and 18 months of age compared to healthy infants. They also found there were higher counts of the species *Firmicutes* such as *Clostridium* and less *Bacteroides* [55], contrasting with previous studies that showed a lack of *Bacteroides* was associated with reduced microbial diversity [54].

So far the evidence on the association between food allergy and gut microbiota is limited. A small Spanish prospective case-control study examined the fecal microbiota of infants at the point of diagnosis of IgE-mediated cow’s milk protein allergy (CMPA) and after 6 months treatment with extensively hydrolyzed formula. They were compared with non-allergic controls. At diagnosis there was no difference in the percentage of bacterial species present in the feces of CMPA infants and controls, but there was a higher overall bacterial count in the allergic infants. Six months post diagnosis the allergic infants had higher proportions of *Lactobacilli* and less *Bifidobacteria* compared to controls [56]. These results contrast with previous studies as the gut bacteria appeared to be more numerous in the CMPA infants compared to controls. However these results were limited as they were based on bacterial culture alone.

Another study by Alderberth *et al*. found no significant association between development of IgE-mediated food sensitization, eczema and stage of fecal microbe colonization [57]. There have been no studies examining microbiota composition prior to the onset of food allergy or utilizing deep sequencing technology for microbiota analysis.

## 3. What is the Evidence that Specific Environmental Factors are Associated with both (a) the Composition of Gut Microbiota, and (b) Risk of Food Allergy?

A variety of factors in the modern environment have been shown to influence the composition of the gut microbiome. These include mode of delivery, antibiotic exposure, infant feeding practices, farming environment and differences between the developed and developing world. The link with food allergy is substantially less clear (Table 1).

**Table 1:** Summary of the associations between specific environmental factors and gut microbiota and food allergy (levels of evidence taken from NHMRC Evidence Hierarchy) [58].

Environmental Factors	Affect Gut Microbiota	Affect Food Allergy Risk
Mode of Delivery	Consistent Level 3 (2) Evidence [59,60,61]	Conflicting Level 3 (3) Evidence [62,63,64,65,66,67,68,69,70]
Antibiotic Exposure	Consistent Level 4 Evidence [71,72]	Limited Level 4 Evidence [66,68,69,73]
Infant Feeding/Diet	Strong Level 3(2) Evidence [59,60]	Conflicting Level 4 Evidence [74]
Farming Environment	Limited Level 4 Evidence [75]	No Evidence
Developing vs. Developed World	Consistent Level 3(2) Evidence [13,76]	Limited Level 4 Evidence [77]

### 3.1. Mode of Delivery

Caesarean-section rates are higher in the developed world compared to the developing world. The USA has witnessed a dramatic increase in the proportion of infants who are delivered by Caesarean-section from 5% of births in 1970 to 31% of births in 2010 [78]. In Canada Caesarean-section rates are more than 25% of total births while in sub-Saharan Africa they range from 4.1% to 16.8 % [60,79].

The fetal gut is believed to be essentially sterile [80] (although recent work has suggested that there may bacteria present in meconium of newborns [81]). In a vaginal birth the baby is colonized by maternal commensal bacteria from the maternal birth canal and gut [82], so the bacterial colonization pattern of the neonate resembles that of the maternal birth canal [49,83]. By six months of age anaerobic species dominate the gut flora, a period which coincides with solid food introduction in most populations [84]. By the age of 2 years the infant gut will have developed an adult type pattern but full development may take several more years [49]. Caesarean-section bypasses the birth canal and in elective sections membranes may only be broken at the time of surgery with the baby delivered through a sterile surface. The gut bacteria lacking in a baby delivered by Caesarean-section include *Bacteroides*, *E-shigella* and *E. coli* [60] and the baby may have higher levels of *Clostridium difficile* [59]. In developing countries *Enterobacteriae* colonization in babies delivered by Caesarean-section tends not to be delayed though this is likely attributable to poorer hygiene standards [85]. The altered gut microbiota pattern associated with Caesarean section can last several years as a Finnish study in 2009 showed lower levels of *Clostridium* in seven year old children delivered by cesarean section compared to those delivered vaginally [61].

The evidence for a direct link between Caesarean-section and food allergy is conflicting. A recent meta-analysis presented at the American Academy of Asthma Allergy and Immunology reported children born to atopic mothers by Caesarean-section were more likely to be sensitized to milk, egg or peanut at 2 years of age [62]. This association appeared to be independent of the mother’s allergy status. However the link with challenge or exposure proven food allergy was not investigated. Concordantly, several observational studies in Europe found a positive association for Caesarean-section and an increased risk of food allergy, especially cow’s milk allergy. In a number of studies the relationship between mode of delivery and risk of food allergy was stronger if the mother was atopic [63,64,65,66,67]. By contrast, in the only population based study to use challenge proven food allergy as the primary outcome, Koplin *et al.* found no evidence for an association between egg allergy and Caesarean-section in Australia [68]. Similarly, a retrospective study from the USA found no difference between mode of delivery and food allergy [69], and a recent Finnish study on children up to 4 years was unable to demonstrate an association between Caesarean-section and development of food allergy [70]. In summary, evidence for an association between mode of delivery and gut microbiota is not reproduced in the limited studies done investigating the association between mode of delivery and food allergy.

### 3.2. Antibiotic Exposure

There is strong evidence that early life exposure to antibiotics influences an infant’s gut microbiota. In the developed world up to 35% of mothers and their babies depending on hospital policy will receive intrapartum antibiotics [86]. Reasons include group B *Streptococcus* infection, maternal pyrexia during labor, postnatal sepsis or prematurity. The most frequent recipients of antibiotics are premature infants in the neonatal intensive care unit. In addition to being resident in a more hygienic environment than standard hospital wards premature infants are also more likely to have been delivered by Caesarean-section than term infants which may confound data. With a prolonged neonatal stay gut colonization may be delayed [87]. The preterm neonatal gut in infants delivered less than 33 weeks gestation is dominated initially by coagulase negative *Staphylococcus* and *Enterobacteriae* [72]. Use of antibiotics have been associated with lower counts of *Bifidobacterium* in the neonatal gut within the first month of life [71].

The evidence regarding the association between exposure to antibiotics and risk of food allergy is again conflicting. A large Finnish study found that perinatal exposure to antibiotics in infants was associated with an increased risk of cow’s milk allergy, as defined by elevated serum specific IgE, positive skin prick test or open challenge test with disappearance of symptoms after cow’s milk elimination from diet [73]. In contrast, a retrospective USA study found no association between food allergy and peri-natal antibiotics [69]; the Australian Healthnuts study found no association between antibiotic use and development of egg allergy [68]; and the Europrevail birth study found no association between maternal antibiotic use in pregnancy and the development of food allergy in infants [66]. Of note, prematurity itself does not appear to confer an increased risk of food allergy [88].

### 3.3. Infant Feeding Practices

Infant feeding influences the composition of the gut microbiota. An infant may be breastfed, formula fed or experience a combination of the two. Adlerberth *et al.* have reviewed evidence indicating the gut microbiota pattern of a breastfed infant showed little difference compared to a formula fed infant [59]. It had been previously suggested that *Lactobacilli* and *Bifidobacterium* counts tend to be higher in breastfed babies but in most studies over the last 30 years no significant differences have been found. However in a recent small Canadian study of 24 infants *Clostridium difficle* levels were significantly lower in breastfed infants compared to the formula fed infants [60]. Certain species of *Clostridium* are well recognized as pathological organisms in older children and adults but the significance of the *Clostridium* genus in neonates is unknown. In addition the infants who were formula fed had a more diverse gut microbiota compared to breastfed infants [60]. Another small study looking at the gut metagenome in breastfed *versus* formula fed infants suggested breastmilk promotes a positive interaction between the mucosal immune system and the gut microbiome through intestinal gene expression [89]. Breastfeeding was also found to be associated with a more diverse gut microbiota. It has been suggested that small mass oligosaccharides found in human breast milk specifically promote the colonization of the gut with a “healthy microbiota”, such as *Bifidobacterium* [90]. The type of infant formula ingested may have an effect on the gut microbiota. A study of 18 infants with cow’s milk protein allergy compared a period of feeding with lactose free extensively hydrolyzed formula, to a period of feeding with lactose containing extensively hydrolyzed formula. The omission of lactose was associated with substantially lower fecal *Bifidobacteria* and *Lactobacilli* as well as reduced concentrations of fecal short chain fatty acids [91]. Another study evaluated the relationship between whey intake and gut microbiota colonization during early infancy by comparing fecal specimens from infants who were fed either high whey containing infant formula, low whey containing formula, or breastmilk. There was no difference in bifidobacterial counts between groups, however *Clostridia* was less prevalent in the breastfeeding group compared to the formula group [92]. Fluorescent *in situ* Hybridization (FISH) was the molecular technique used to analyze the microbiota in this study.

Intake of complex carbohydrates during infancy also affects the composition of the gut microbiota. In a study of nineteen infants intake of a formula high in complex carbohydrates was associated with a higher concentration of butyric acid (a short chain fatty acid) in the feces compared to the comparison group [93]. In addition, intake of the formula high in complex carbohydrates was associated with elevated fecal secretory IgA levels supporting the influence of complex carbohydrates on gut microbiota composition as secretory IgA production is associated with the presence of *Bifidobacterium* in the gut [94]. 

Allergy prevention is hypothesized to be associated with greater microbial diversity but previous studies have been unable to produce conclusive evidence that exclusive breastfeeding is protective against allergic disease [74]. Human breastmilk is a rich source of secretory IgA in early postnatal life [95]. It has been shown that intake of secretory IgA in breastmilk in the first year of life may be associated with a reduced risk of atopic dermatitis [96]. It has also been shown in another trial that maternal avoidance of cow’s milk during breastfeeding was associated with a reduced level of *β*-lactogloblulin specific IgA in the breastmilk and a higher rate of cow’s milk allergy in their offspring [97].

The relationship between infant feeding particularly breastfeeding and food allergy is difficult to disentangle as there are both ethical and feasibility barriers to conducting randomized trials. 

### 3.4. Farming Environment

The “hygiene hypothesis” articulated by Strachan in the late 1980s proposed that exposure to infection in early life through larger family sizes was protective against allergic disease [7]. Subsequent work, however, suggests that, rather than infectious diseases *per se*, the early life microbial experience in general is a key determinate of immune development and risk of allergic disease. It would seem logical to assume that exposure to a less hygienic environment throughout childhood such as a farm would promote the acquisition of a more diverse human gut microbiota. However there is only circumstantial evidence to support this assumption. Indeed, one study in 2007 comparing children from several European countries found children from farming backgrounds had less gut microbial diversity compared to non-farming children from the same geographical area [75].

For allergy the most compelling evidence comes from studies demonstrating that farming environment is associated with a reduced risk of allergic disease and that most of this effect appears to be related to microbial exposure. For example, the European GABRIELA and PARSIFIL studies found that children raised in farming environments had decreased rates of allergic disease, and that around 90% of this effect could be attributed to microbial exposure, rather than other factors in the farming environment such as sun exposure [10,11,12]. This may, in part relate to higher and more efficient Treg cell numbers at birth among infants from farming environments [98].

Therefore, while the protective effect of farming environment has been shown for asthma and allergic sensitization, studies into the relationship between farming environment and risk of food allergy are both insufficient and conflicting. 

### 3.5. Developing vs. Developed World

Birth in a higher income country is associated with delayed gut colonization, reduced microbial diversity [13] and reduced turnover of bacterial strains in the infant gut [76]. There are a range of factors that may be involved, including hygiene practices and diet [99]. Concordantly, birth in a high income country is associated with an increased risk of allergic disease [100]. Further, it has been observed that migration from a developing to a developed country is associated with an increased risk of allergic disease, but only if migration occurs during the first years of life [77]. In a recent Australian study it was found that migration from South East Asia to Australia was associated with an increased risk of eczema in the infants of the migrants [101]. 

## 4. Implications and Challenges for Future Studies

### 4.1. Evaluating Gut Microbiota Composition

The gut microbiota is a complex entity making it difficult to characterize in a meaningful way. Early attempts to characterize the gut microbiota used fecal culture-based techniques. However, less than 30% of the total fecal bacteria are identifiable using culture based techniques [102] providing a limited picture. Recent advances in microbiota measurement technology now provide greater information on the breadth of microbiota present generating enormous amounts of information that allow comparison of overall patterns/signatures which may be more relevant and useful in understanding complex biological responses. Most approaches are based on analysis of 16S rRNA [103], a common component of bacterial ribosomes containing variable regions that can be used for phylogenetic identification. 

One of the first and simplest molecular techniques was the analysis of Terminal Restriction Fragment Length Polymorphisms (TRFLP), following digestion of 16S rRNA amplicons using restriction enzymes [104]. TRFLP analysis generates profiles in which more abundant groups of bacteria give higher peaks, and greater diversity results in a greater number of peaks. TRFLP, however, provides a relatively non-specific and blunt picture because it does not differentiate to the species level.

More recently, phylogenetic microarrays, such as HITChip [105], have been developed to provide a semi-quantitative profile of species-specific extracted DNA or 16S rRNA amplicons that are fluorescently labeled [106]. Phylogenetic microarrays have the advantage over TRFLP techniques of providing phylogenetic identification. It is also a qualitative technique that can identify particular species, although cross hybridization can result in multiple probes hitting single targets [107]. The main use of phylogenetic microarray is to compare microbial populations between different hosts.

Sequencing is a DNA extraction based method that requires information from the full 16S rRNA gene to be successful. This can then be compared to a standardized gene database to allow evaluation of specific microbiota [108], although the approach has relatively high cost and low throughput.

Next Generation Sequencing (NGS) has been developed as a lower cost method based on multiple sequencing of DNA templates. NGS utilizes massive parallel sequencing of partial 16S rRNA gene amplicons, and consequently can run at 2,000 times the rate of earlier sequencing techniques, enabling less abundant bacteria to be detected [102]. NGS is used mainly to compare differing microbiota populations.

Metagenomics is the newest method used to measure gut microbiota. Metagenomics provides data regarding the function of the microbiota in addition to the genetic diversity, and was the technique used in the recent Human microbiome project. Metagenomics is achieved by random fragmenting of DNA which reconstructed to create a continuous sequence. This approach facilitates the recognition of microbial genes that enhance bacteria-host interactions [21]. 

### 4.2. Analysis of Gut Microbiota Data

#### 4.2.1. Microbial Diversity

It is important to recognize that, rather than being a specific biological entity, “microbial diversity” is variably a constructed metric encompassing the relative abundance distribution of distinct types of organisms and bacterial families. Other terms used to describe microbial diversity are “richness” and “evenness”. For example, if a sample had a few dominant species present at similar levels it would be described as having low richness, and high evenness. Microbial diversity is a semi-quantitative measure, that can be used to make comparisons between individuals with and without a given exposure or outcome of interest. Differences in fecal microbial diversity have been linked to several human diseases [109]. Recent studies have shown that breastfeeding may be associated with less microbial diversity. This is an unexpected finding as breastfeeding is thought to be protective against allergy and allergy prevention is associated with greater microbial diversity, and previous studies had suggested that breastfeeding may promote gut microbial diversity [60,110]. 

In order to make the comparison of the microbial diversity of different samples relevant mathematical models have been used to provide a quantitative measure. The Shannon index is a measure of entropy (biodiversity) of the sample and the uncertainty of the sampling outcome [111]. The other commonly used model, the Simpson diversity index is an examination of the probability that taking two readings from the same sample will produce an equivalent result [112]. Each produces a numerical value used to compare different samples.

#### 4.2.2. The Metabolic Signature

An alternative or complementary approach to evaluating the relationship between the composition of gut microbiota and disease is to focus on the microbiota metabolites rather than the microbiota themselves. The potential advantages of focusing on the metabolic signature include the fact that a wide variety of organisms may exert their biological effect via a more finite group of metabolite-host interactions; and further, that the metabolic signature is reflective of both the composition of the gut microbiome and the substrate (*i.e.*, diet) available to these organisms. For example, there is mounting interest in the relationship between dietary intake of fermentable fibers, the production of short chain fatty acids (SCFA), and risk of allergic disease [113].

The SCFA are produced by anaerobic bacteria including the *Firmicutes* species [114] and have been recognized as one of the most important gut microbiota products as they are involved in several gut functions that maintain a healthy state. These include immune and inflammatory responses. For example, GPR43 is protein receptor present on immune cells and colonic epithelium that binds SCFA’s. The SCFA acetate is the only known ligand of GPR43. In a mouse model, it was shown that clinical and histological evidence of colitis was reduced after introduction of dietary acetate, but this benefit was absent in GPR43 knockout strains [115]. A study in 2000 by Bottcher *et al.* showed that SCFA levels including butyric acid was lower in children with asthma, atopic dermatitis or food allergy [116]. Measurement of SCFA levels in stools including butyric acid may provide the metabolic signature to evaluate the gut microbiota in healthy and diseased states.

### 4.3. What Types of Studies are Required?

The opportunity to employ newer techniques such as next generation sequencing to provide a more detailed analysis of the gut microbiota has led to a vast array of information available about the bacteria species that comprise the gut microbiome. There is now a clear need to grasp this opportunity through future studies that incorporate the longitudinal assembly of data regarding the determinants of gut microbiota composition and risk factors for food allergy. Collection of maternal stool prior to delivery and infant stool on regular occasions during the first years of life will add to this data collection. Previous studies have relied on either retrospective parent reported allergy or specific IgE to identify allergy status which has lacked objectiveness. Prospectively, determination of primarily food sensitization and subsequently challenge proven IgE-mediated food allergy status will provide an ideal cohort for gut microbiota analysis at an early life stage which has been lacking in previous studies. There are, however, important logistical and feasibility considerations for such studies. The ability to prospectively assemble a large cohort and retain the majority of participants throughout the study period is a major challenge. In addition the standardization, collection and storage of numerous biospecimens require dedicated trained staff and involves potentially high costs.

The need for longitudinal studies determining the potential relationship between gut microbiota and food allergy is well established. In the event of a positive association the next steps are trials of intervention where there are already several promising approaches. These include dietary manipulation, direct supplementation with specific microbiota metabolic products (e.g., delivery of acetate or butyrate to the bowel), modification of determinants of microbiota composition (e.g., perinatal antibiotics for group B *Streptococcus* prophylaxis) and fecal transplant to humans. 

## 5. Conclusions

Food allergy rates in children have rapidly increased in both the developed and developing world and the possible causes merit investigation. The microbiome that occupies the human gut and its role in health and disease has become the target of intense scrutiny in recent years and there is evidence of its importance in autoimmune disease, obesity and allergic disease in general. It has been demonstrated that in early life the gut microbiota influence immune development and balance of Treg cells which may increase the risk of food allergy. Environmental factors that may affect gut microbiota patterns in early life include obstetric interventions in pregnancy, antibiotic exposure, infant feeding practices and a modern westernized lifestyle. Epidemiological studies examining the influence of these factors on gut microbiota have yielded consistently strong evidence for positive associations. In parallel, studies examining the same factors and the risk of food allergy have revealed at best weak associations. While there has only been to date limited examination of the relationship between gut microbiota and food allergy in humans, several studies on gut microbiota and other allergic disease such as eczema have yielded positive associations. An altered pattern of gut microbiota colonization in the gut in the early life period may directly increase the risk of food allergy in children. Prospective longitudinal cohort studies that obtain biospecimens at regular intervals for analysis and provide robust outcomes such as challenge proven food allergy are required. Subsequently, knowledge of the relationship between early life gut microbiota and allergic disease may facilitate development of novel prevention and treatment strategies.
